# Effects of temperature and repeat layer spacing on mechanical properties of graphene/polycrystalline copper nanolaminated composites under shear loading

**DOI:** 10.3762/bjnano.12.65

**Published:** 2021-08-12

**Authors:** Chia-Wei Huang, Man-Ping Chang, Te-Hua Fang

**Affiliations:** 1 Department of Mechanical Engineering, National Kaohsiung University of Science and Technology, Kaohsiung 807618, Taiwan

**Keywords:** dislocation, graphene/Cu, molecular dynamics, shear, self-healing

## Abstract

In the present study, the characteristics of graphene/polycrystalline copper nanolaminated (GPCuNL) composites under shear loading are investigated by molecular dynamics simulations. The effects of different temperatures, graphene chirality, repeat layer spacing, and grain size on the mechanical properties, such as failure mechanism, dislocation, and shear modulus, are observed. The results indicate that as the temperature increases, the content of Shockley dislocations will increase and the maximum shear stress of the zigzag and armchair directions also decreases. The mechanical strength of the zigzag direction is more dependent on the temperature than that of the armchair direction. Moreover, self-healing occurs in the armchair direction, which causes the shear stress to increase after failure. Furthermore, the maximum shear stress and the shear strength of the composites decrease with an increase of the repeat layer spacing. Also, the shear modulus increases by increasing the grain size of copper.

## Introduction

Graphene is a monolayered hexagonal thin film composed of sp^2^-bonded carbon atoms and has extraordinary properties for applications in nanoelectronics [1–6]. However, because of the two-dimensional structure, graphene is limited as a structural material [7–8]. In addition, research on composite materials found that nanocomposites can provide better performance than a single material [9]. Therefore, three-dimensional graphene composites have been developed in recent years, which exhibit excellent properties in various fields, such as supercapacitors, integrated electrodes, catalysis, and sensors [10–13]. Furthermore, the interaction between graphene and matrix materials directly affects the mechanical properties of composites [14]. The van der Waals force between graphene and metals can increase the strength of composites [15–16]. Thus, graphene–metal composites are regarded as important three-dimensional composites.

Numerous studies on the enhancement of mechanical properties of graphene–metal composites have been reported. Chu et al. demonstrated that with 10 vol % graphene nanoplatelets (GNPs) in a copper matrix the in-plane tensile strength was increased by 26% [17]. Li et al. used graphene nanoplatelets decorated with Ni nanoparticles as addition in a Cu matrix. These Ni-GPL/Cu composites exhibited a 42% increase in ultimate tensile strength (UTS) over that of pure Cu, with only 0.8 vol % of Ni-GPLs [18]. Jiang et al. demonstrated that a pristine graphene (PG)/Cu composite showed a 90% enhancement of yield strength, and 81% increase of compression strength compared to pure copper [19]. Graphene is regarded as a reinforcement component in composites; it has been confirmed that even random graphene fragments can improve the mechanical properties of composites. However, it is challenging to tailor the mechanical properties by controlling the proportion of graphene since the mechanical behavior of graphene composites is associated with the microstructure [20–21]. Therefore, the design of ordered graphene composites and the development of optimized microstructures is a major issue to study.

In 2013, Kim et al. synthesized metal–graphene nanolayered (MGNL) composites consisting of alternating metal (copper or nickel) layers and graphene monolayers [22]. Since then, a series of studies on three-dimensional nanolaminated graphene–metal composites have been carried out. Several studies revealed that the graphene interface provides a barrier to impede the propagation of dislocations, and simultaneously enhances the mechanical properties of the composites [22–25]. The competition between interface and matrix in deformation and failure of a nanolayered polycrystalline Cu–graphene material has been reported [26]. In 2018, Weng et al. reported the potential strengthening mechanism of graphene/copper composites under compression. The results show that the incorporation of single-layer graphene enhances the mechanical properties of the composites [27]. Shin et al. indicated that there are several factors that would affect the reinforcement of the composite material, including the geometric/spatial characteristics of the reinforcement and the interface [28].

In this work, we focus on temperature and microstructure effects on graphene/polycrystalline copper nanolaminated (GPCuNL) composites. As mentioned above, the microstructure plays a crucial role for the mechanical behavior of GPCuNL composites. Thus, in the present study, molecular dynamics (MD) simulations of shear loading with different metal layer spacings and grain sizes are conducted. Besides, the temperature effect is also considered since metal lattices become disordered and soften with an increase in temperature [29–30]. Gayk et al. revealed the relationship between different potentials and the mechanical properties of carbon materials [31]. In this study, graphene with zigzag and armchair chirality is considered while simulating different properties of graphene monolayers and copper layers, including stress–strain curve variations, the distribution of von Mises stress, the evolution of dislocations, the out-of-plane displacement, and the self-healing behavior of the graphene layer. The obtained results provide valuable information for the design of three-dimensional graphene composites.

## Computational Method

**Figure 1:**
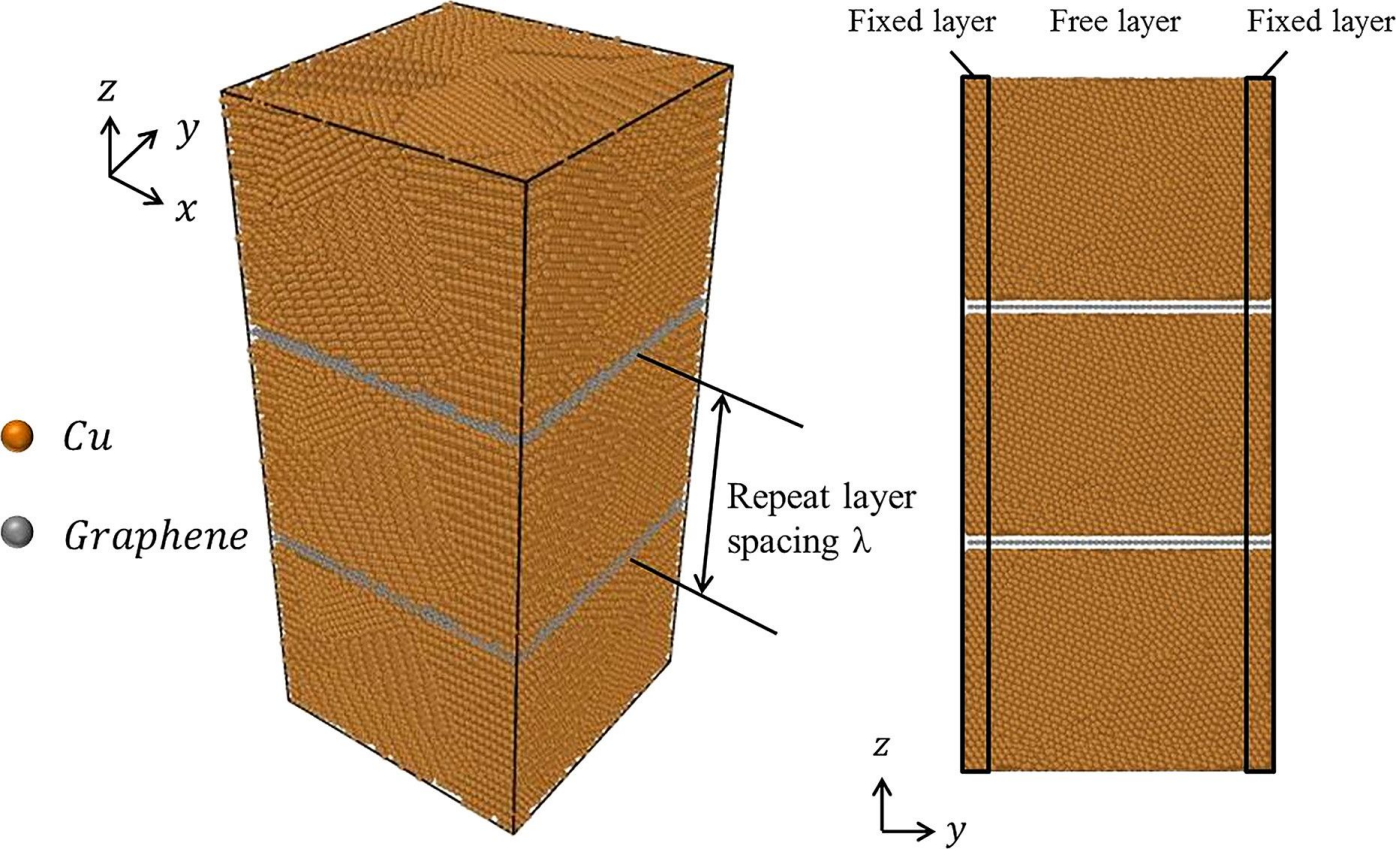
Schematic of GPCuNL composite.

**Figure 2:**
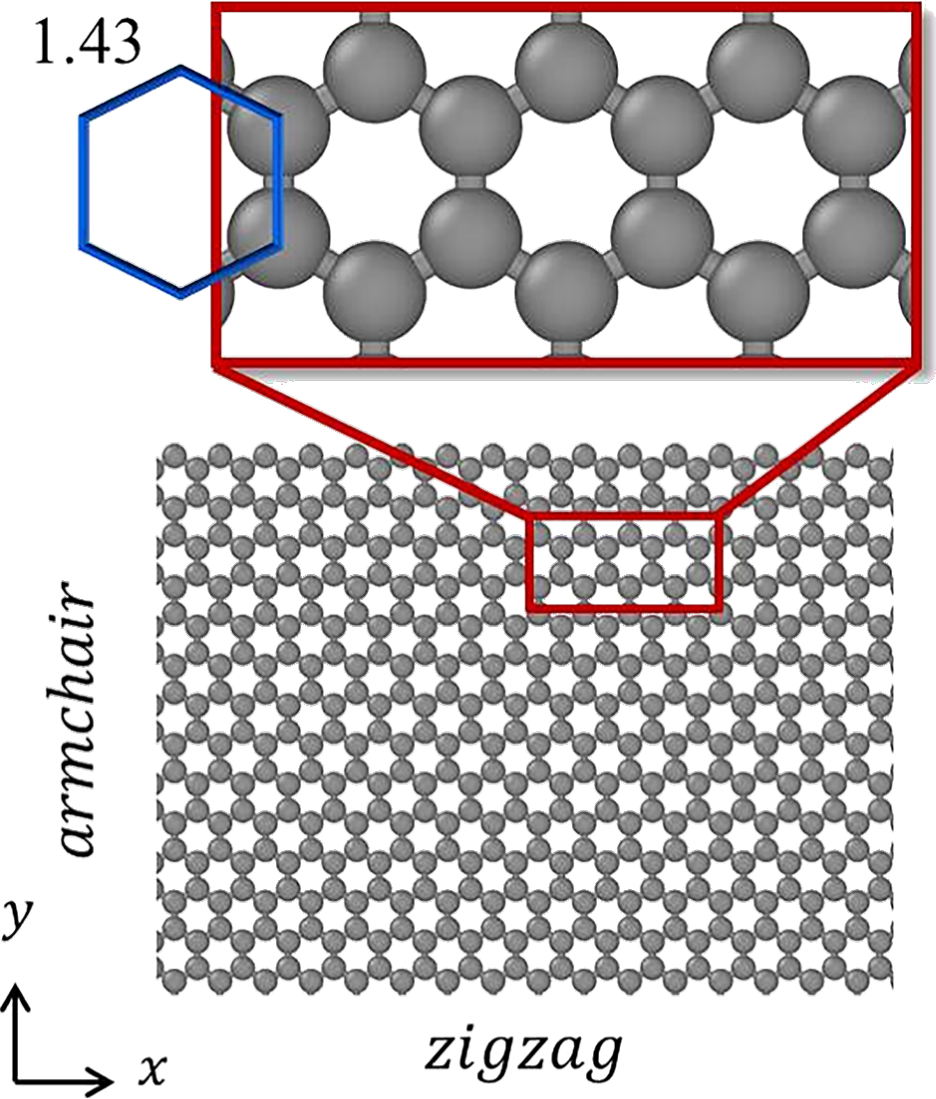
Two different graphene chiralities and the bond length of graphene.

A model of the graphene/polycrystalline copper nanolaminated (GPCuNL) composites was built using the open source software AtomSK [32]. As shown in Figure 1, the dimensions of the nanolaminated composites model are set to 10 nm × 10 nm × 22 nm and the layer thickness is fixed at 0.5 nm. Sizes of 8 nm × 8 nm × 22 nm, 10 nm × 10 nm × 22 nm, and 12 nm × 12 nm × 22 nm were tested during the construction of the simulation models. Different sizes of the model were tested to find the optimal size for good simulations at minimal computational time and to determine if finite-size effects arise from the model dimensions. A size of 10 nm × 10 nm × 22 nm of the model was found acceptable. The average grain size *D* is calculated by 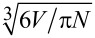, where *V* is the total volume of polycrystalline copper and *N* is the number of grains [33]. Periodic boundary conditions are used in three axial directions to simulate the infinite material models. The crystal orientations of the polycrystalline copper are [100], [010], and [001] along the *x*-, *y*-, and *z*-directions, respectively. According to a previous report, the C–C bond length in the graphene monolayer was set to 0.143 nm [34], as shown in Figure 2.

The simulations were carried out using the large-scale atomic/molecular mass parallel simulator (LAMMPS), with a time step of 1 fs and a strain rate of 1.25 × 10^8^ s^−1^. The interaction between Cu atoms is described by the embedded atom model (EAM) potential. The adaptive intermolecular reactive empirical bond order (AIREBO) potential describes the forces between C atoms, the cutoff radius is chosen as 10.2 Å [31]. The interactions between C and Cu atoms are calculated by Lennard-Jones (LJ) potentials, using parameters of 0.019996 eV and 3.225 Å [35]. The simulation system uses an isothermal–isobaric (NPT) ensemble to reach equilibrium by a Nose–Hoover thermostat for 100000 time steps. The shear loading is performed by applying a consistent velocity to the composites along the *x*-direction. The grain size *D* of the simulation model ranges from 4.24 to 9.43 nm. Deformation and dislocation evolution of the model are analyzed and visualized using the Open Visualization Tool (OVITO).

## Results and Discussion

### Effect of temperature and chirality

**Figure 3:**
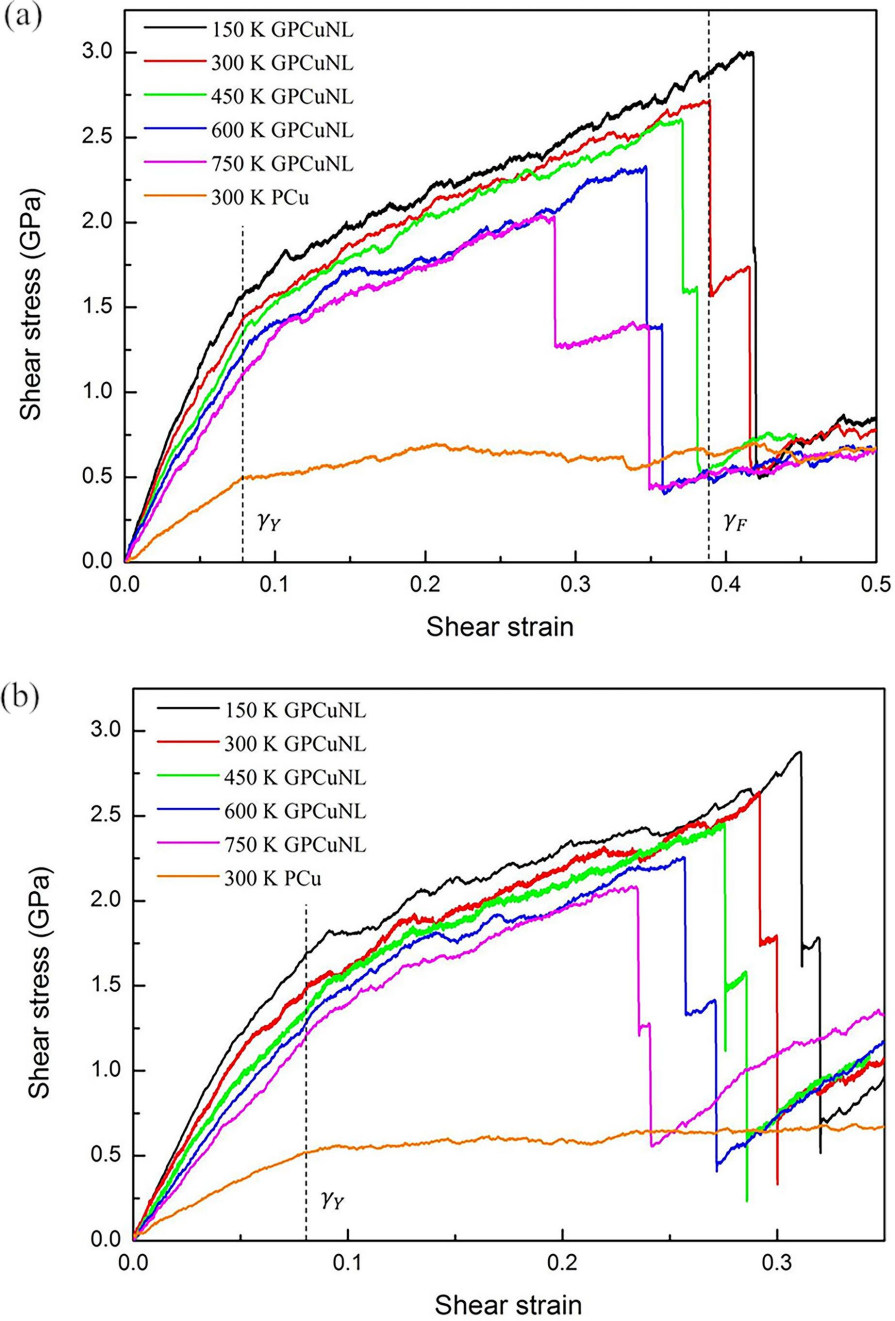
Stress–strain curves of (a) zigzag and (b) armchair GPCuNL composites at different temperatures.

The stress–strain curves of GPCuNL composites and polycrystalline copper (PCu) at different temperatures and with different graphene chirality are presented in Figure 3, where yield and failure strain are defined as γ_Y_ and γ_F_, respectively. As shown in Figure 3, the shear stress of the composites decreases with an increase in the temperature for both zigzag and armchair directions. The stress–strain curve of GPCuNL composites can be divided into three stages, which are γ_Y_ > γ, γ_F_ > γ > γ_Y_, and γ_F_ ≤ γ. During the first stage (γ_Y_ > γ), the shear stress and shear strain are linearly proportional, which represents an elastic deformation of the composites. During the second stage (γ_F_ > γ > γ_Y_), the shear strain still increases with an increase in shear stress and the composites begin to deform plastically. During the third stage (γ_F_ ≤ γ), the shear stress decreases rapidly at a specific strain. In the case of the GPCuNL composite at 300 K, the shear stress drops suddenly when the strain is 0.39. Because the two layers of graphene do not fracture at the same time, a plateau appears in the curve followed by another drop at γ = 0.415, indicating another graphene failure. Furthermore, the shear stress of the composites is reduced to a constant value of about 0.5 GPa, which is close to the maximum shear stress of PCu. It is obvious that the shear strength of GPCuNL is above five times that of PCu, indicating that the embedded graphene can effectively improve the overall strength of the composites.

**Figure 4:**
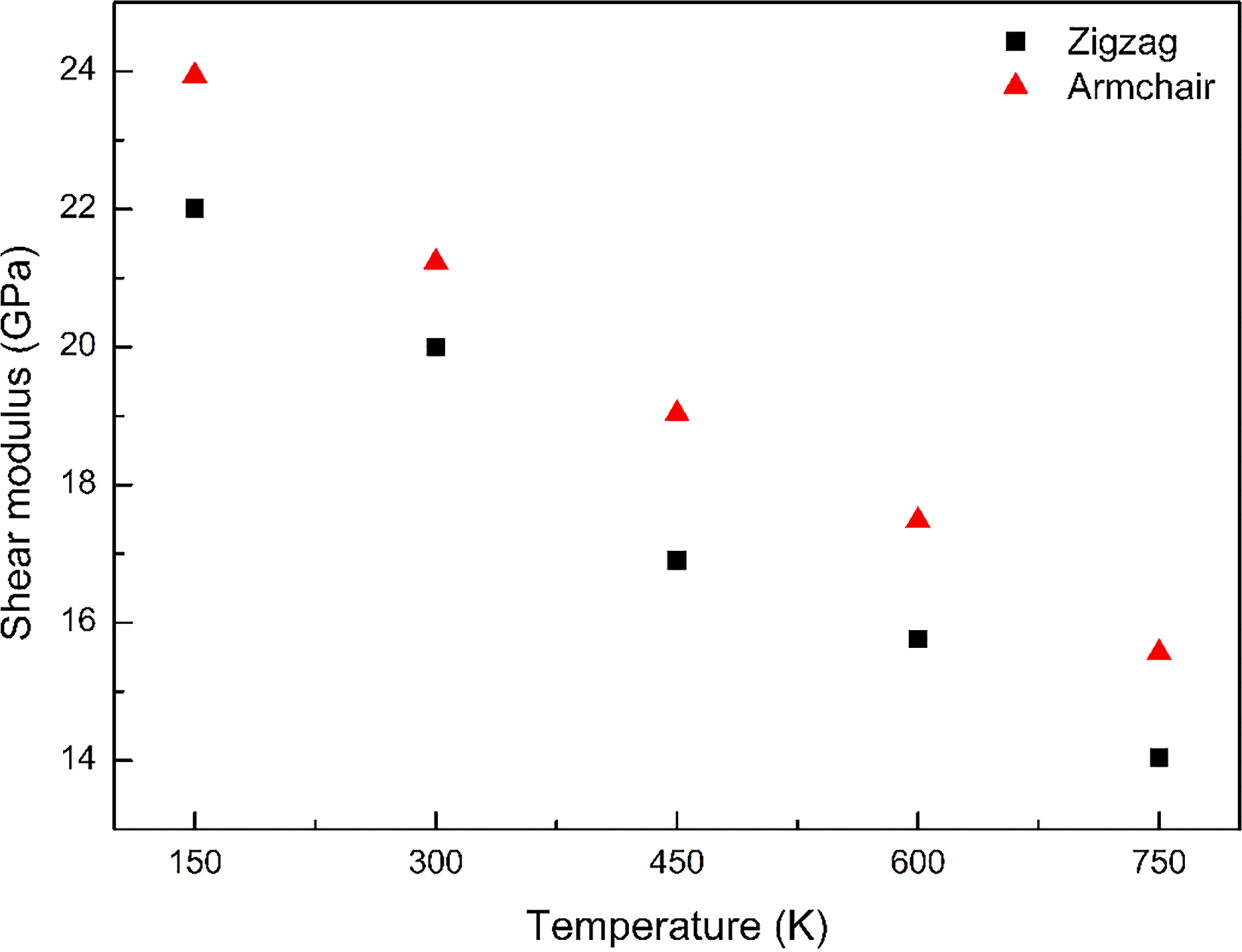
The shear modulus of GPCuNL composites at different temperatures and with different graphene chiralities.

Figure 4 shows the shear modulus of GPCuNL composites at different temperatures and with different graphene chiralities. With the increase of temperature, the shear modulus of the zigzag and armchair GPCuNL composites significantly decreased. Moreover, the reduction rate of the shear modulus of the zigzag direction is higher than that of the armchair direction, that is, 36.2% and 35% from 150 to 750 K, respectively.

**Figure 5:**
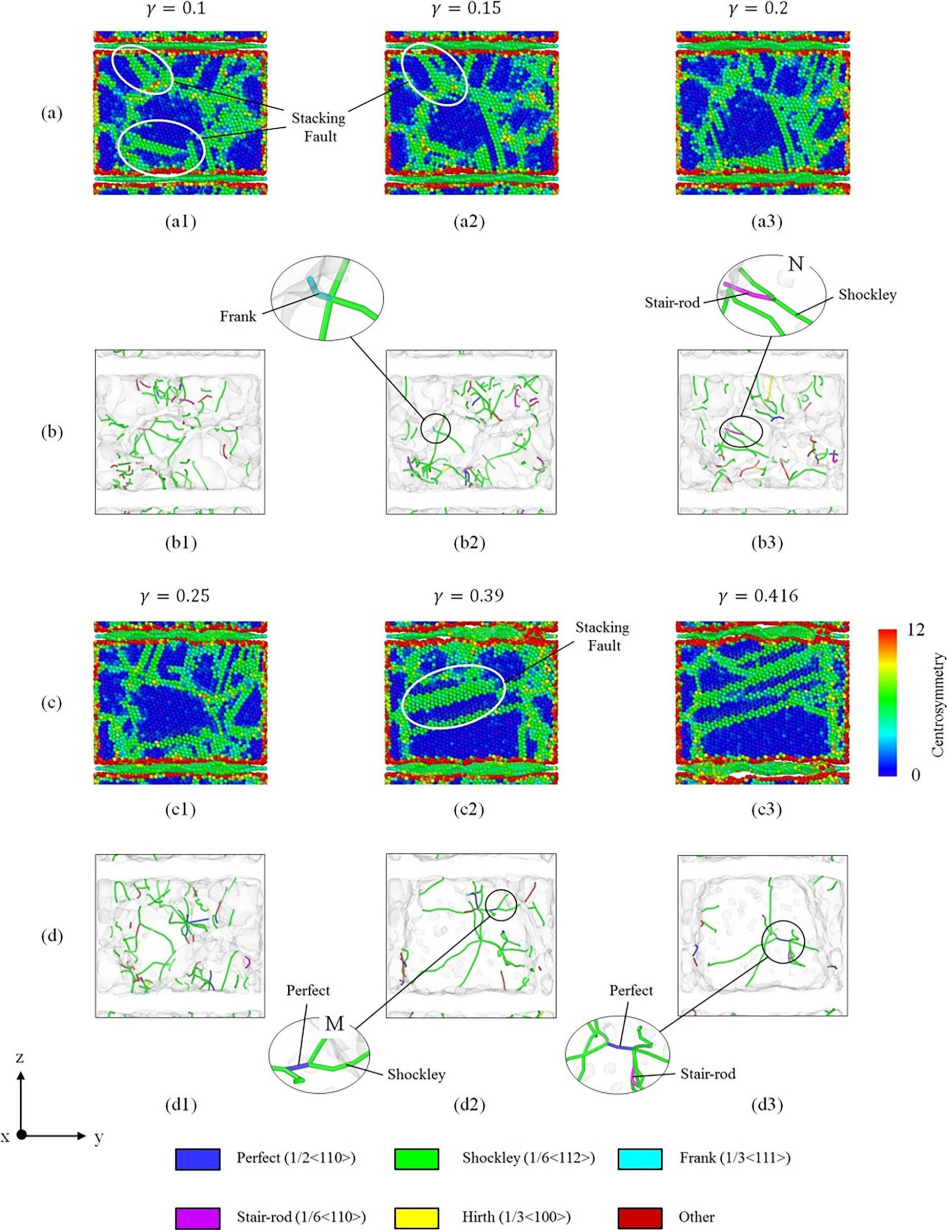
(a, c) Cross-sectional view of the CSP analysis of the GPCuNL composites and (b, d) DXA analysis of the dislocation evolution of the GPCuNL composites along the zigzag direction at 300 K.

Stacking faults and dislocations are crucial factors for the deformation of crystalline materials. The centrosymmetry parameter (CSP) and the dislocation extraction algorithm (DXA) analysis are used to evaluate the behavior of dislocations and stacking faults of the GPCuNL composites under shear loading. Figure 5a,c provides a cross-sectional view of the CSP analysis, and Figure 5b,d presents the DXA analysis of the dislocation evolution along the zigzag direction at 300 K. Under shear loading stacking faults occur in the polycrystalline Cu (Figure 5a3,c2) with a phase transition from the face-centered cubic (FCC, blue) to the hexagonal closest packed (HCP, green part) structure. Besides, several kinds of dislocations are formed in the polycrystalline Cu, as shown in Figure 5b,d, that is, perfect, Shockley, Hirth, stair-rod, and Frank dislocations with corresponding Burgers vectors of 1/2<110>, 1/6<112>, 1/3<100>, 1/6<110>, and 1/3<111>, respectively. For face-centered metals, the Shockley partial dislocations are important since they are associated with material slip [36]. Shockley partial dislocations are formed by the separation of perfect dislocations [37]. The magnified image (M) in Figure 5d2 elucidates the formation of Shockley partial dislocations is according to the equation

[1] $$\frac{1}{2}\left[0\bar{1}\bar{1}\right]\to \frac{1}{6}\left[\bar{1}\bar{2}\bar{1}\right]+\frac{1}{6}\left[1\bar{1}\bar{2}\right].$$

In addition, the relationship between the stair-rod dislocation and the Shockley partial dislocation is shown in the magnified image (N), where it is evident that a stair-rod dislocation (1/6⟨011⟩) dissociates into the 1/6⟨12−1⟩ and 1/6⟨−1−12⟩ Shockley partial dislocations as illustrated in the following equation [38]:

[2] $$\frac{1}{6}\left[011\right]\to \frac{1}{6}\left[12\bar{1}\right]+\frac{1}{6}\left[\bar{1}\bar{1}2\right].$$

**Figure 6:**
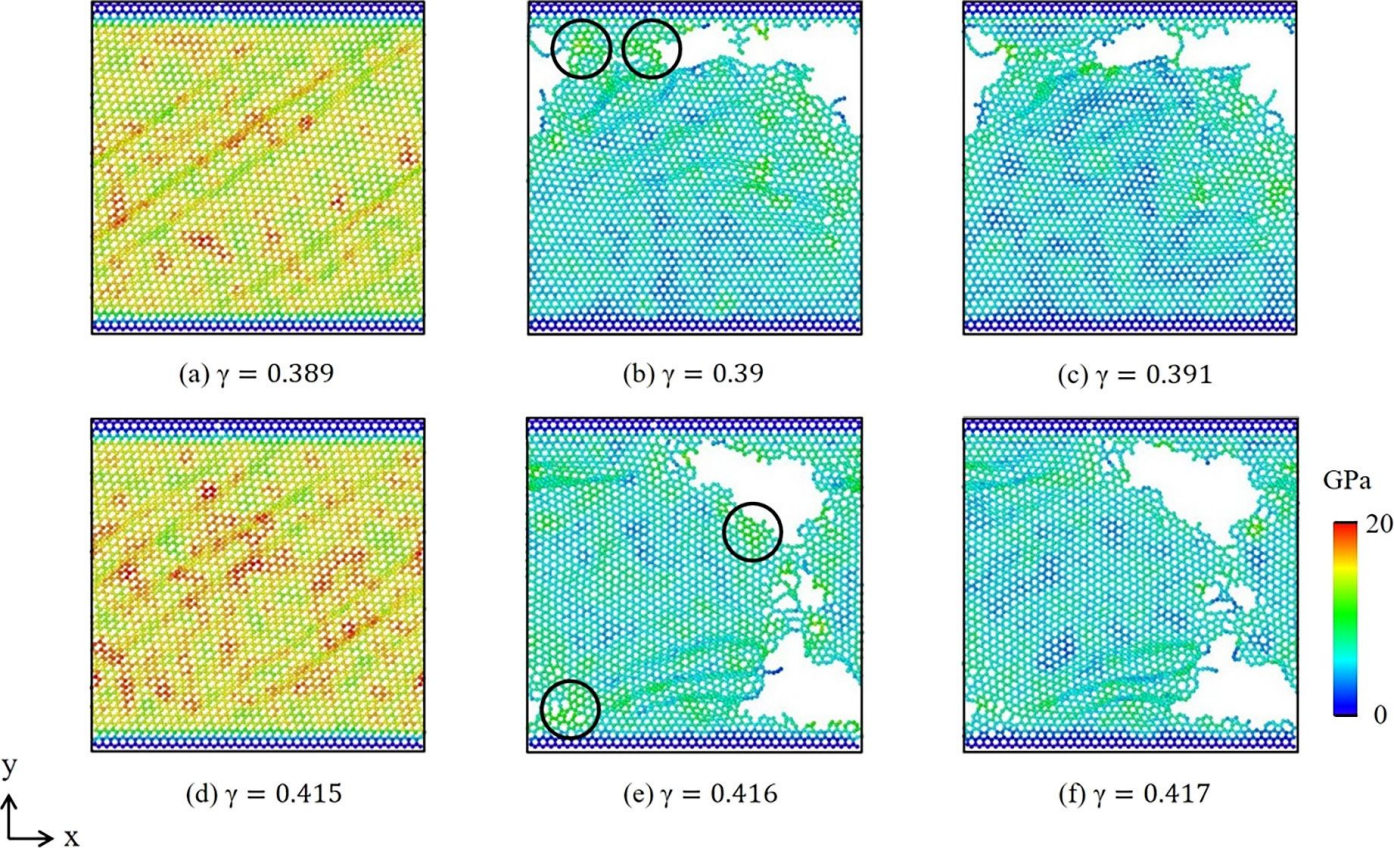
(a–f) Von Mises stress of graphene under shear loading along the zigzag direction at 300 K. (a–c) Top graphene sheet, (d–f) bottom graphene sheet.

Figure 6a–f shows the von Mises stress in graphene along the zigzag direction at 300 K. The results show that the top and the bottom graphene sheets are fractured sequentially. After the top graphene sheet fails, it can be clearly observed that the stress begins to concentrate at the bottom graphene sheet. Until the failure of the bottom graphene sheet at γ = 0.416. After that, the higher stress appears around the cracks. Moreover, if the loading increases continuously, the crack promptly expands and shows a zigzag shape for the zigzag direction.

**Figure 7:**
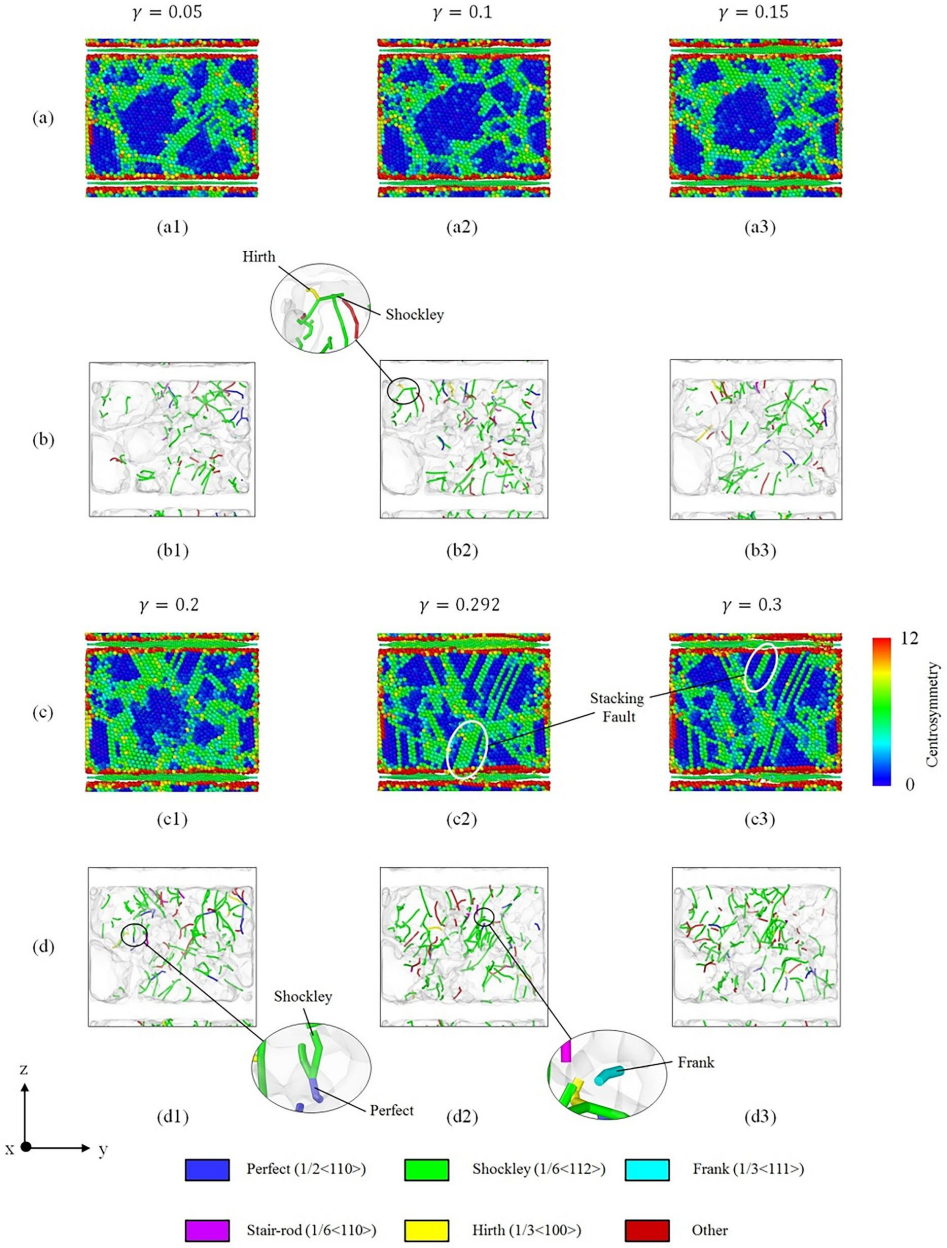
(a, c) Cross-sectional view of the CSP analysis of the GPCuNL composites and (b, d) DXA analysis of the dislocation evolution of the GPCuNL composites along the armchair direction at 300 K.

**Figure 8:**
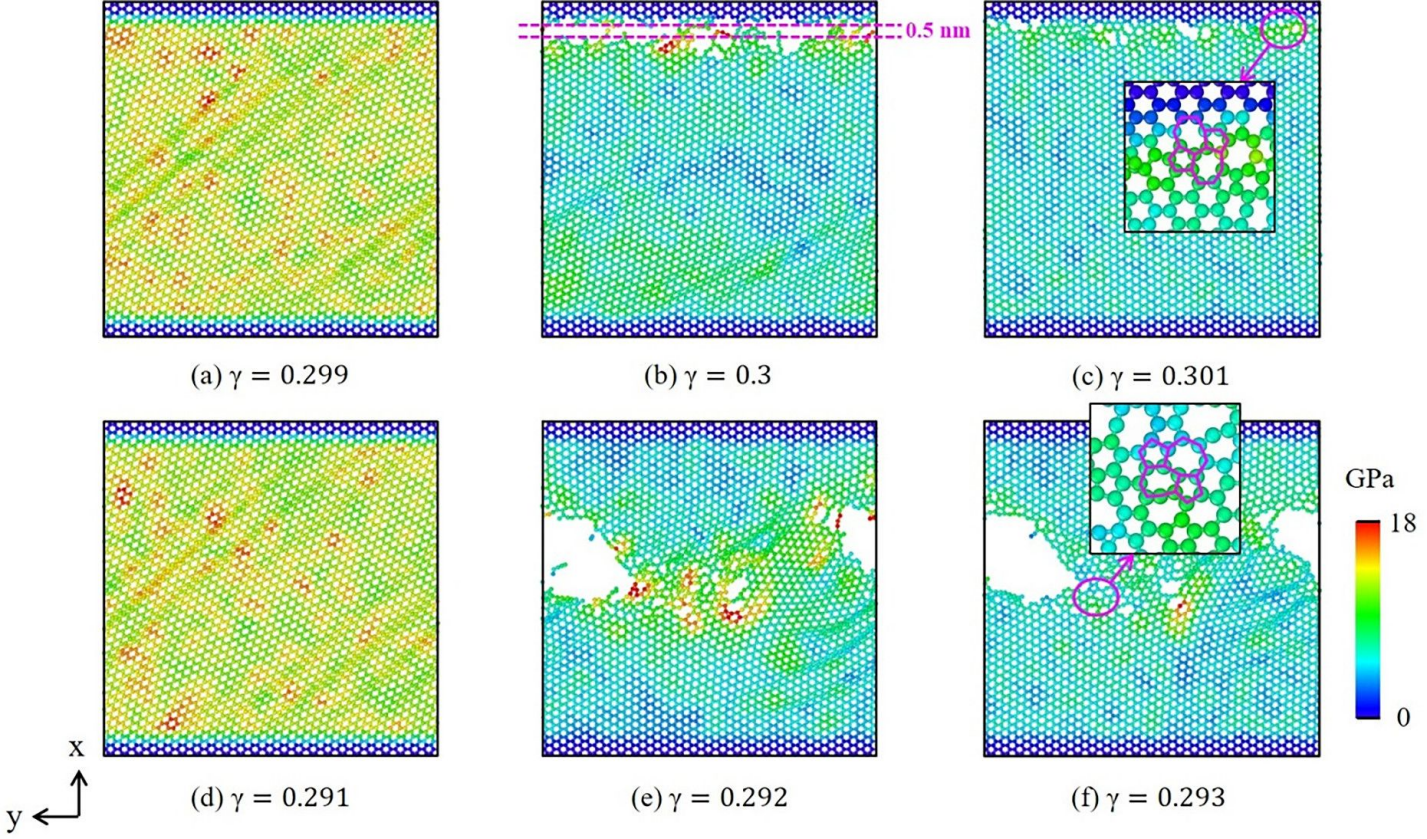
(a–f) Von Mises stress of graphene under shear loading along the armchair direction at 300 K. (a)–(c) Top graphene sheet, (d–f) bottom graphene sheet.

**Figure 9:**
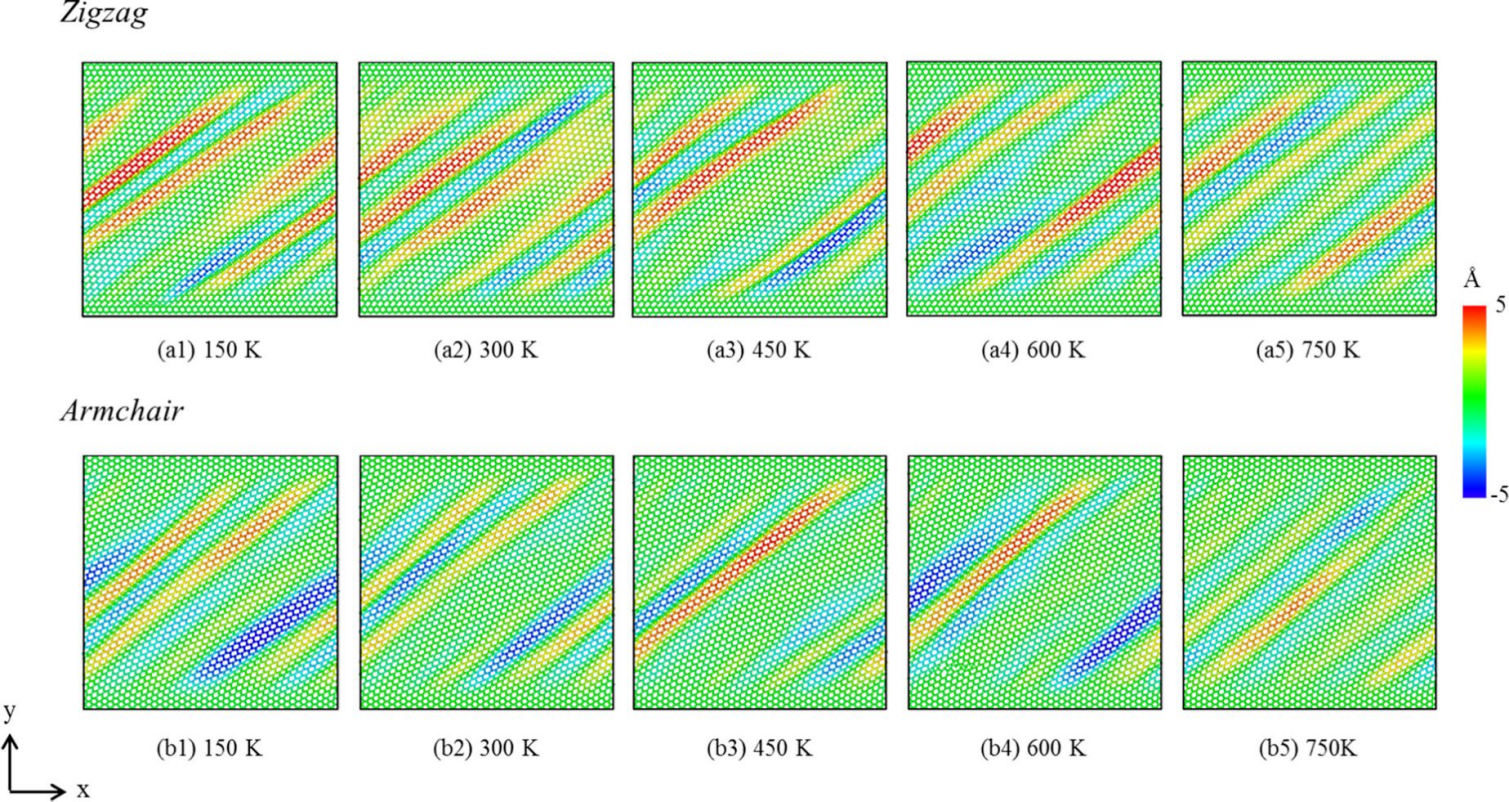
Out-of-plane displacement of graphene at different temperatures. (a1–a5) Zigzag graphene, (b1–b5) armchair graphene.

Figure 7a,c provides a cross-sectional image of the CSP analysis, and Figure 7b,d presents the DXA analysis of the dislocation evolution in the GPCuNL composites along the armchair direction at 300 K. The CSP images show that stacking faults form at the interface and increase under shear loading. The DXA analysis shows that perfect dislocations and Shockley partial dislocations appear under varying strain. Figure 8 shows the von Mises stress in graphene along the armchair direction at 300 K. As shown in Figure 8b and Figure 8c, for the armchair direction, the self-healing phenomenon at the crack is observed after fracture. Self-healing is caused by the bonding of carbon atoms in graphene. At γ = 0.3, there are dangling bonds between the carbon atoms at the crack, which are unsaturated bonds with unstable energy [39]. If the width of the crack is ≤0.5 nm, the chemical binding energy is negative, and attractive forces cause the self-healing of the carbon atoms [40]. As shown in Figure 8c, the carbon atoms are rearranged to form a pentagon–heptagon (5–7–7–5) structure, which is known as the Stone–Wales defect structure [40]. It can be seen as the result of a 90° rotation of the C–C bonding, which transfers four hexagons into two pentagons and two heptagons [41–42]. After self-healing, the strength of graphene is maintained but reduced slightly due to the Stone–Wales defects. As shown in Figure 3, the shear stress rises again after failure. Moreover, Figure 9 shows the out-of-plane displacement of graphene at different temperatures. For zigzag and armchair directions, the wrinkle fluctuations of graphene at different temperatures are in the range of ±5 Å, which is caused by the constrains of the copper layers. Furthermore, if the shear loading increases continuously, in the case of armchair direction, graphene will rapture along the crack.

Since the zigzag direction exhibits better mechanical properties than the armchair direction at different temperatures, the subsequent studies are all considering the zigzag direction.

### Effect of repeat layer spacing

**Figure 10:**
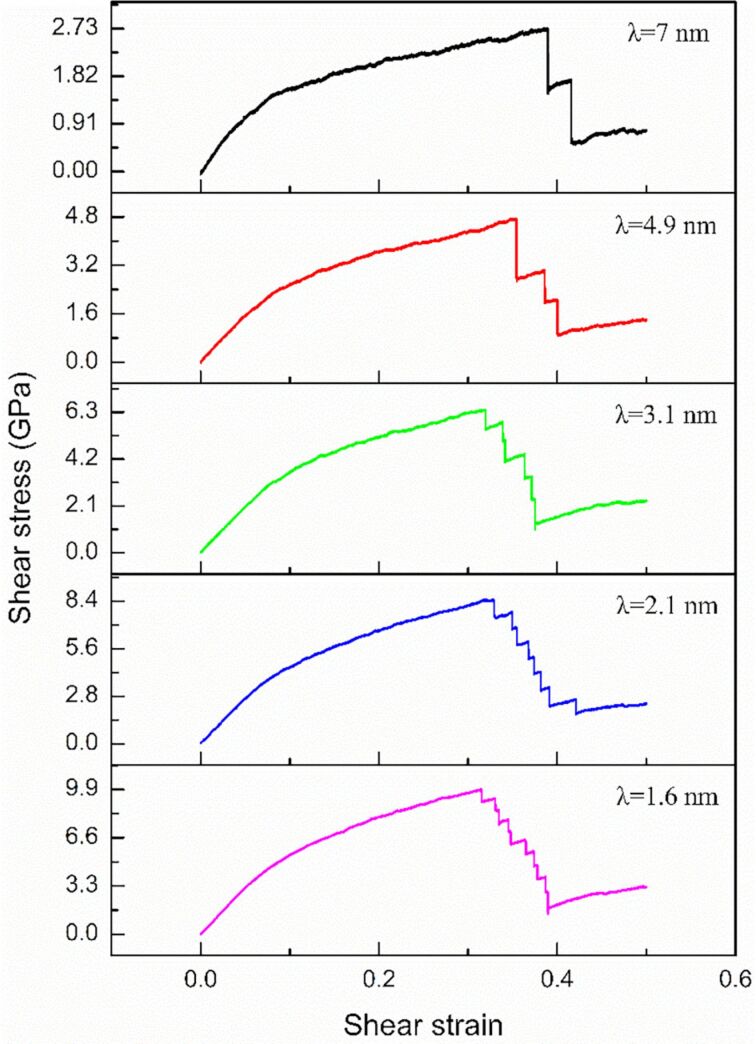
Stress–strain curves of zigzag graphene/Cu composites with different repeat layer spacings.

**Figure 11:**
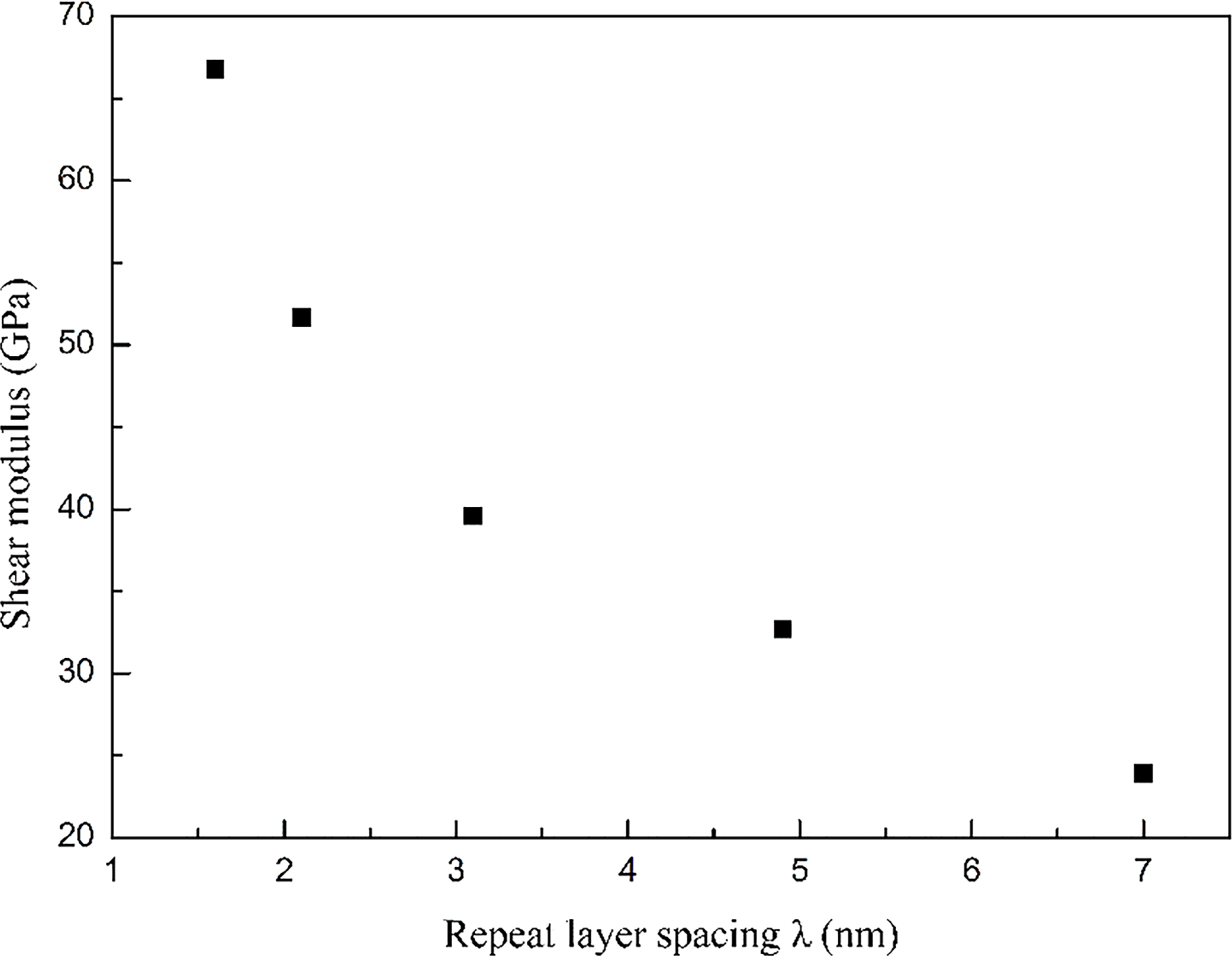
The shear modulus of zigzag GPCuNL composites with different repeat layer spacings.

To estimate the effect of the layer spacing λ, two layers (λ = 7 nm), four layers (λ = 4.9 nm), six layers (λ = 3.1 nm), eight layers (λ = 2.1 nm), and ten layers (λ = 1.6 nm) are used to examine the effect of changing the distance between zigzag graphene sheets on the mechanical properties of the GPCuNL composites. Figure 10 depicts the stress–strain curve of zigzag graphene/Cu composites with different repeat layer spacings at 300 K. The maximum shear stresses are 2.74, 4.72, 6.42, 8.46, and 9.91 GPa corresponding to λ = 7, 4.9, 3.1, 2.1, and 1.6 nm, respectively. The results show that the maximum shear stress value increases with decreasing repeat layer spacing. After the stress reaches its maximum value, the curves drop due to the fracture of graphene, and many stairs form in the curves. The number of the stairs increases with an increase of the layer numbers. Therefore, it can be concluded that graphene and copper do not fracture simultaneously but layer by layer, which can effectively avoid the instant fracture of the composites and reinforce the plasticity under shear loading. Figure 11 shows the shear modulus of zigzag graphene/Cu composites with different repeat layer spacings at 300 K. The shear modulus decreases with an increase in layer spacing. For λ = 1.6 nm, the shear modulus is 67 GPa; and for λ = 7 nm, the shear modulus is only about 24 GPa. which shows that changing the layer spacing has a significant effect on the mechanical properties.

**Figure 12:**
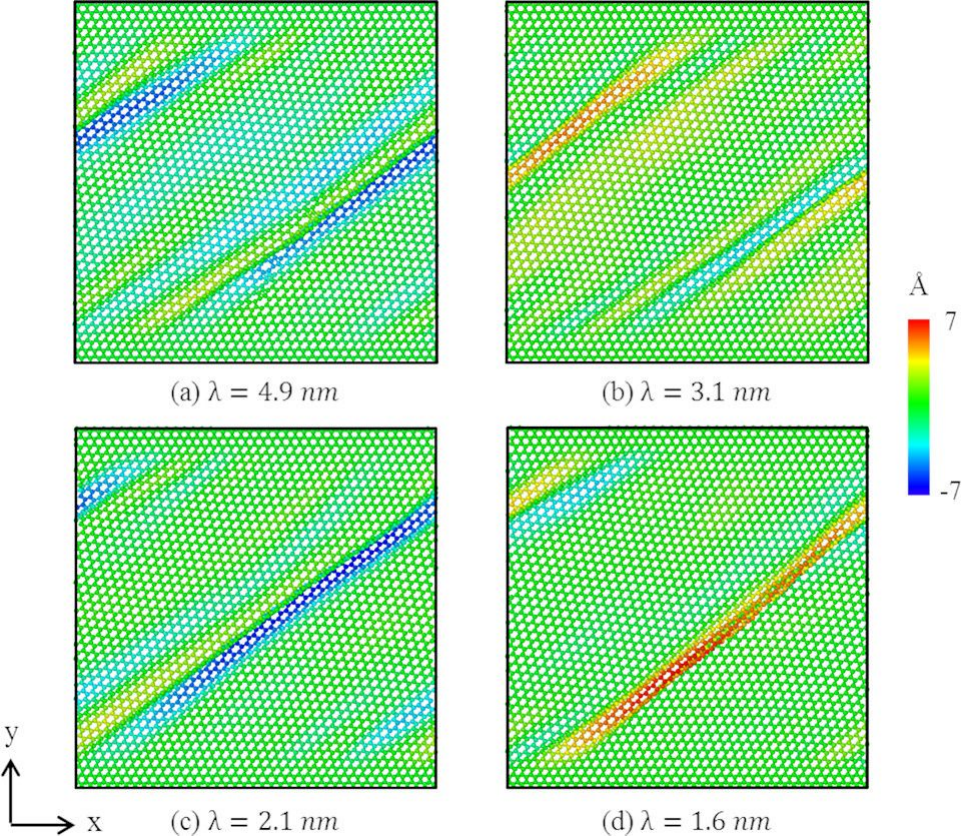
The out-of-plane displacement of zigzag graphene with different repeat layer spacings.

**Figure 13:**
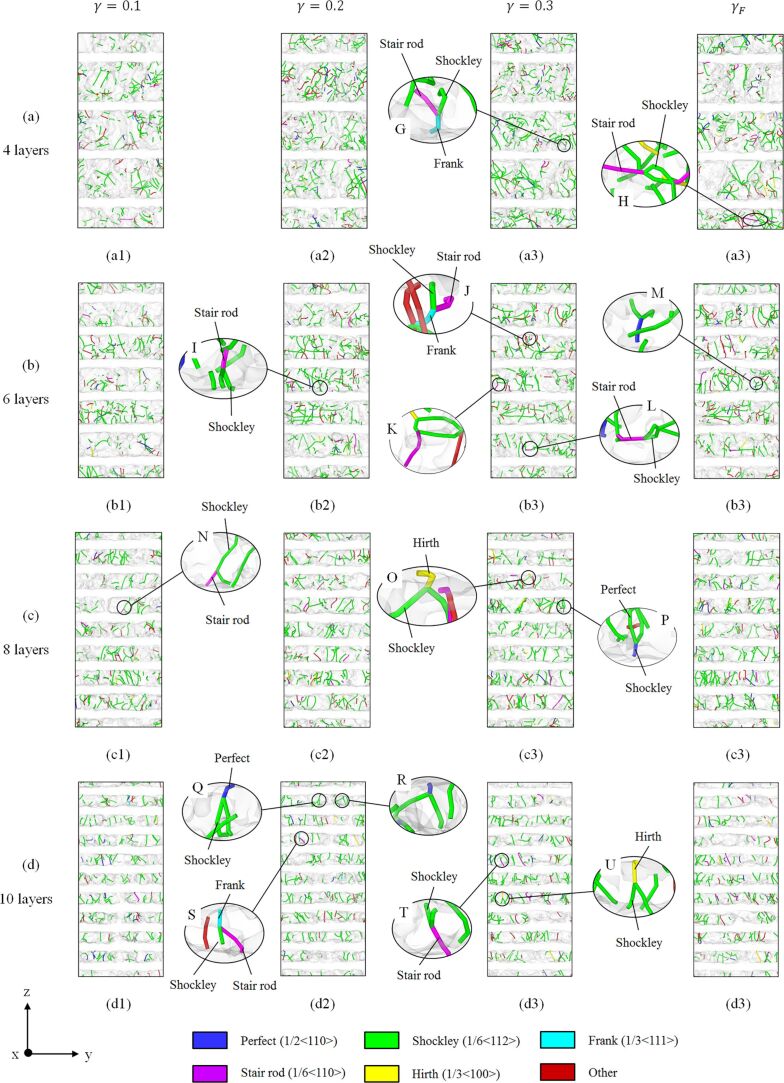
The DXA analysis of GPCuNL composites with different repeat layer spacings at 300 K. The magnified images (M), (P), (Q), and (R) elucidate the formation of perfect dislocations from two Shockley partial dislocations. The magnified images (H), (I), (K), (L), (N), and (T) show the relationship between stair-rod dislocations and Shockley dislocations. The magnified images (O) and (U) show the formation of a Hirth dislocation from two Shockley dislocations with vertical Burgers vectors.

Figure 12 shows the out-of-plane displacement of zigzag graphene with different repeat layer spacings. It can be observed that when λ > 2 nm, the displacement of graphene is in the range of ±5 Å, as shown in Figure 12a–c. By contrast, when λ < 2 nm, a wrinkle with a displacement larger than 7 nm arises, as shown in Figure 12d. Since the copper layer no longer constrains the displacement of graphene, large wrinkles can form [26]. Therefore, when the layer spacing of GPCuNL composites is smaller than 2 nm, instability of the composite structure may occur. Furthermore, Figure 13 shows the DXA analysis of the dislocation evolution of GPCuNL composites with different repeat layer spacings at 300 K. It can be observed that Shockley partial dislocations are the principal dislocations in the composites, which account for 70% of all dislocations.

### Effect of grain size

**Figure 14:**
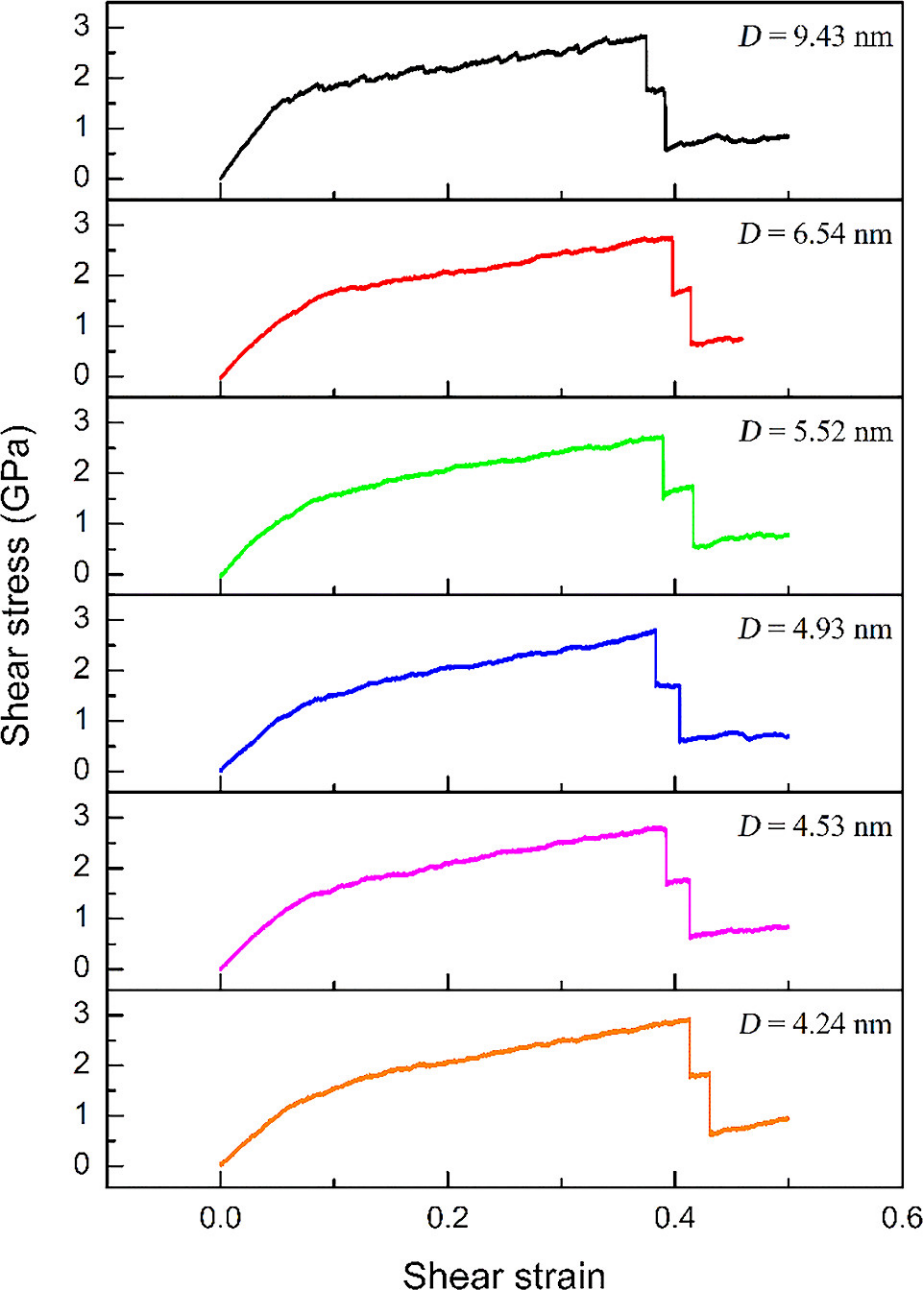
Stress–strain curves of zigzag GPCuNL composites with different grain sizes.

**Figure 15:**
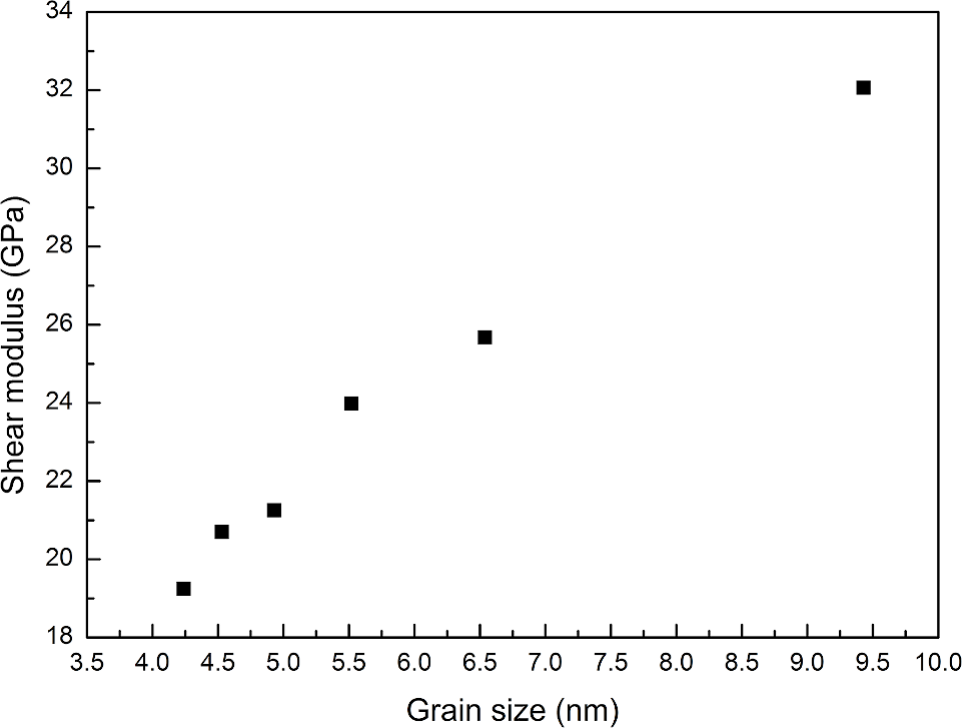
Shear modulus of zigzag GPCuNL composites with different grain sizes.

In order to estimate the effect of grain size (*D*) on the mechanical properties of the GPCuNL composites under shear loading, six different grain sizes in the copper layer are modeled, which are 9.43, 6.54, 5.52, 4.93, 4.53, and 4.24 nm. Figure 14 shows the shear stress–strain relationship of the zigzag GPCuNL composites with different copper grain sizes. The average strain of these specimens is about 0.39 ± 0.02, and the average stress is about 2.79 ± 0.1 GPa. The smallest grain (*D* = 4.24 nm) shows the maximum shear stress and strain. In terms of the shear strength, the Hall–Petch relationship confirms that the strength of the composite specimens will increase with a decrease of the grain size [43–44]. Because of grain boundary strengthening, plastic deformation hardly occurs during the loading process [43–44]. However, when the grain size is less than 10 nm, grain boundary sliding drives the movement of atoms and leads the specimen to deform and become soft. Therefore, the strength of the specimens will decrease as the grain size decreases [45]. Although the 9.43 nm grain size may slightly affect the grain boundary behavior due to size effects, the results do not deviate. The shear modulus of the composite specimens with six different grain sizes is calculated, as shown in Figure 15. The values of the shear modulus decrease with a decrease of the grain size, which is consistent with the above results.

**Figure 16:**
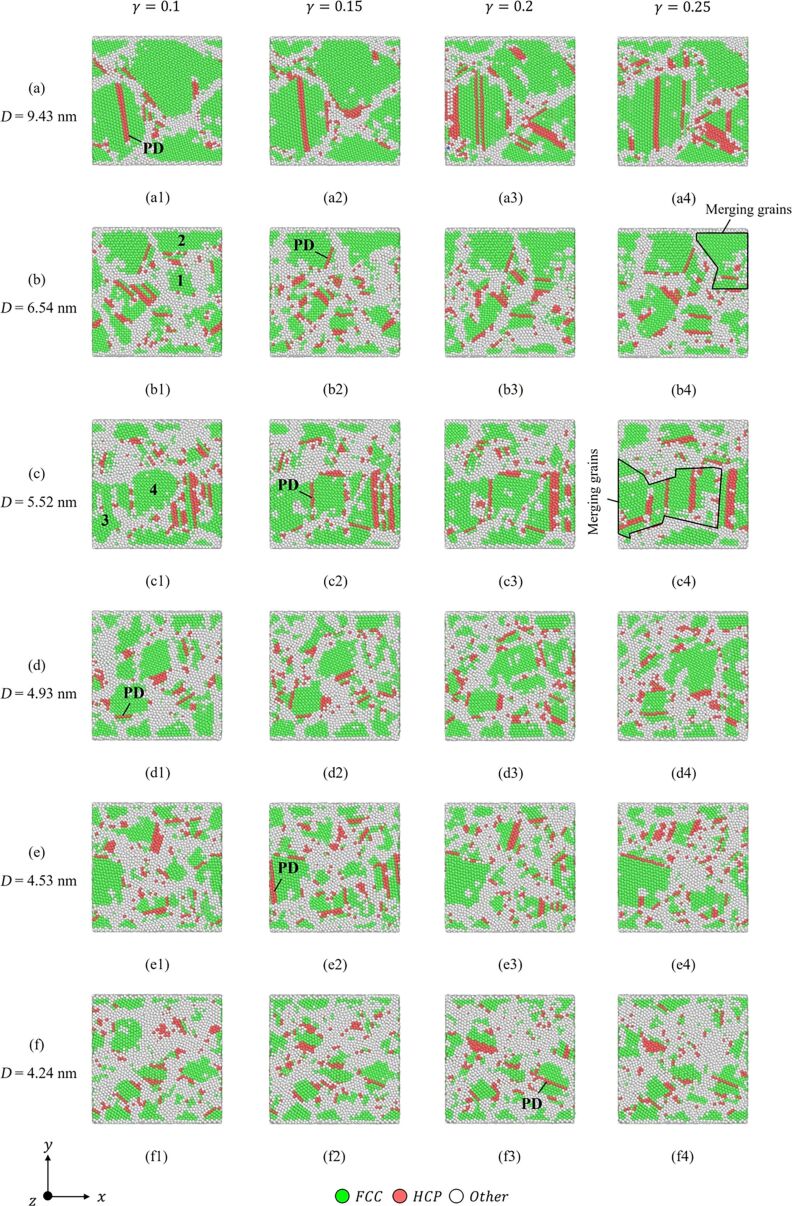
The structural evolution of polycrystalline Cu with different grain sizes. The “PD” symbols represent partial dislocations.

Figure 16 depicts the CNA diagram of different grain sizes under shear loading. The strains are γ = 0.1 (Figure 16a1–f1), γ = 0.15 (Figure 16a2–f2), γ = 0.2 (Figure 16a3–f3), and γ = 0.25 (Figure 16a4–f4). The “PD” symbols represent partial dislocations. It can be observed that dislocations and stacking faults occur in all copper layers. Stacking faults are produced from two adjacent HCP layers. These defects are caused by the release of energy stored in the specimens. The deformation of the polycrystalline structure is mostly affected by the grain boundaries [46–47]. When the dislocations propagate to the grain boundaries, they will cause the grain boundaries to slide and twist. The dislocations may be absorbed by the grain boundaries or diffuse into the grains. Besides, the evolution of grains is also one of the main factors of crystal structure deformation. As shown in Figure 16b1 and Figure 16c1, grains “1” and “2” and grains “3” and “4” will merge with increasing shear strain and form new grains with dislocations and stacking faults, as shown in Figure 16b4 and Figure 16c4. The evolution of grains is due to the local displacement of grain boundaries and atoms adjacent to the grain boundaries, which leads to the diffusion of atoms and the gliding of the dislocations, as shown in Figure 16a1–a4. Besides, the deformation mechanism also depends on the grain size. In the case of large grains (*D* = 9.43 nm), the propagation of dislocations and stacking faults mainly occurs inside the grains, as shown in Figure 16a. These dislocations can interact within the grains to form dislocation networks or can interact with the dislocations in the grain boundaries. Moreover, with an increase of shear loading, these dislocations will be destroyed by other dislocations, or move into the grain boundaries, where they can be absorbed and cause the grain boundaries to expand. In contrast, in the case of small grains (*D* = 4.24 nm), due to the small grain size, dislocations and stacking faults cannot move inside the grains, as shown in Figure 16f.

**Figure 17:**
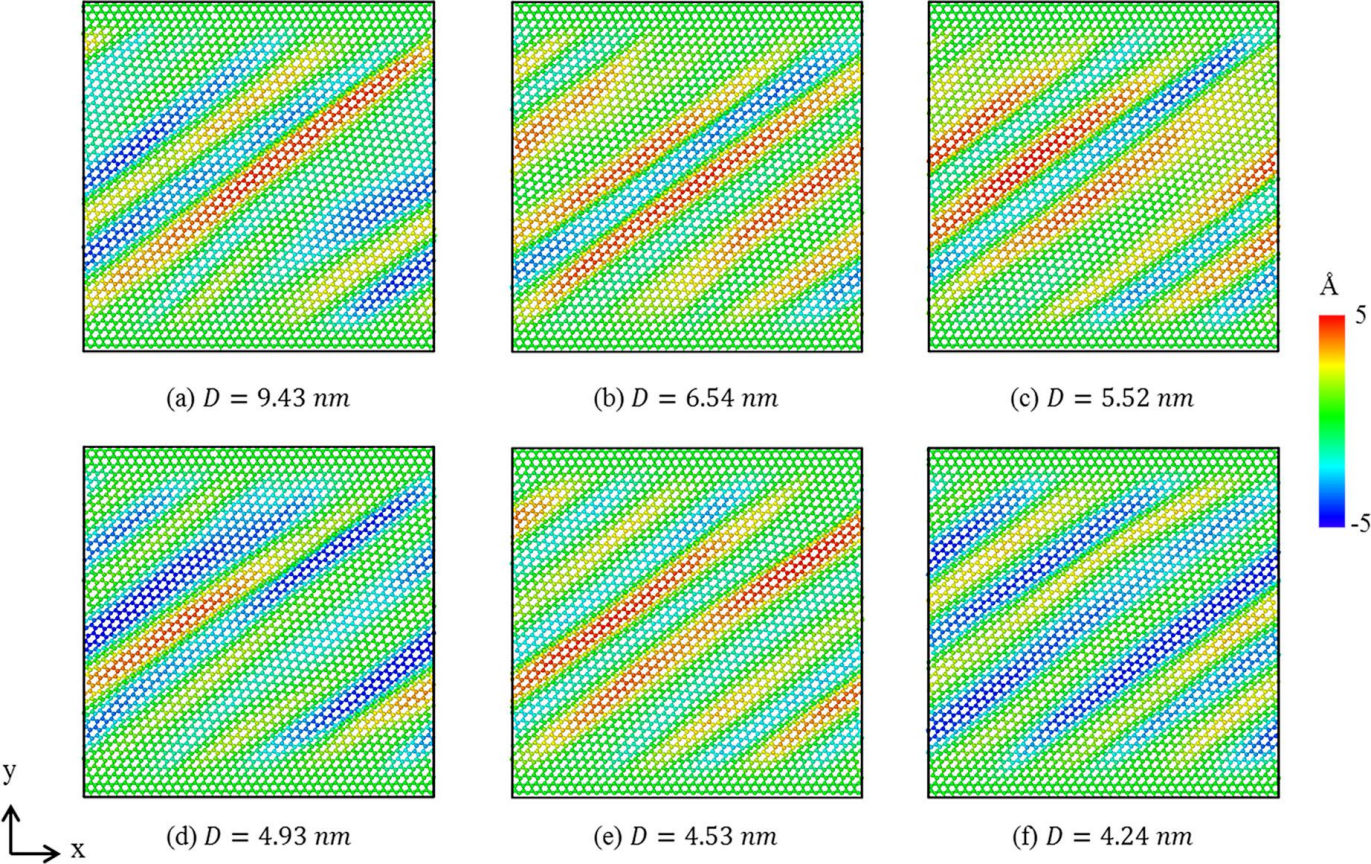
The out-of-plane displacement of zigzag graphene in GPCuNL composites with different grain sizes.

Figure 17 shows the out-of-plane displacement of zigzag graphene in GPCuNL composites with different grain sizes. It can be observed that, for large and small grains, the wrinkle fluctuations produced in graphene are ±5 Å, which is equivalent to the displacement mentioned above for the temperature effect. Therefore, the change of copper grain size will not affect the wrinkles of graphene. Although changing the grain size does not cause many effects on the shear stress and strain of the composites, it is still an important factor in changing the shear strength and the movement of defects of the composite specimens.

## Conclusion

In this study, shear loading of GPCuNL composites is performed using MD simulations. The effects of different temperatures, graphene chirality, repeat layer spacing, and grain size on the deformation behavior of the composites are investigated. The following results are obtained: (1) For the zigzag direction of graphene, the maximum shear stress and strain will decrease with an increase of temperature. After the composite structure is destroyed, the shear stress is reduced to about 0.5 GPa, which is close to the shear stress value of polycrystalline Cu. By contrast, for the armchair direction, the maximum shear stress and strain also decrease with increasing temperature, and the maximum shear stress is lower than that of the zigzag direction. However, because of the self-healing phenomenon of graphene, the shear stress after failure rises again. Therefore, the armchair composite still maintains a certain strength. (2) Regarding the repeat layer spacing, if the layer spacing is λ > 2 nm, the out-of-plane displacement of graphene is in the range of ±5 Å; if λ < 2 nm, graphene is no longer constrained by the copper layer. Moreover, the maximum shear stress increases as the layer spacing decreases. The shear modulus decreases with an increase in the layer spacing. Compared with other effects, the layer spacing affects the mechanical properties of the GPCuNL composite more obviously. (3) Concerning the grain size of Cu, the out-of-plane displacement of zigzag graphene with different Cu grain sizes stays between ±5 Å. Besides, the maximum shear stress shows little difference for different grain sizes. However, the shear modulus increases with increasing grain size. Therefore, the grain size still plays an important role regarding the shear strength of the GPCuNL composites.
